# Retinal and Corneal OCT Results of Patients Hospitalized and Treated in the Acute Phase of COVID-19

**DOI:** 10.3390/jcm13185564

**Published:** 2024-09-19

**Authors:** Edward Wylęgała, Aleksandra Prus-Ludwig, Patrycja Mocek, Tomasz Tomczyk, Bogdan Dugiełło, Andrzej Madej, Bogusława Orzechowska-Wylęgała, Adam Wylęgała

**Affiliations:** 1 Chair and Clinical Department of Ophthalmology, Faculty of Medical Sciences in Zabrze, Medical University of Silesia, 40-760 Katowice, Poland; 2 Department of Ophthalmology, Railway Hospital, 40-760 Katowice, Poland; 3 Ophthalmology Department, I School of Medicine, Katowice, Medical University of Silesia, 40-514 Katowice, Poland; 4 Department of Pharmacology, WSB University, 41-300 Dąbrowa Górnicza, Poland; 5 Department of Pediatric Otolaryngology, Head and Neck Surgery, Department of Pediatric Surgery, Medical University of Silesia, 40-760 Katowice, Poland; 6 Health Promotion and Obesity Management Unit, Department of Pathophysiology, Faculty of Medical Sciences, Medical University of Silesia, 40-752 Katowice, Poland

**Keywords:** COVID-19, optical coherence tomography (OCT), cornea, retina, oxygen therapy, remdesivir, tocilizumab, COVID-19 convalescent plasma therapy

## Abstract

**Objective:** This study aimed to assess changes in the morphology of the retina and cornea in patients treated and hospitalized during the acute active phase of SARS-CoV-2 infection. **Methods:** A total of 24 patients with symptomatic early COVID-19 disease and 38 healthy participants from a control group were enrolled in our study. Among them, 20 received oxygen therapy at flow rates ranging from 1–10 L, while four received high-flow intranasal oxygen therapy (HFNOT). Some patients were treated with other types of therapy, such as Remdesivir, COVID-19 convalescent plasma therapy, or Tocilizumab. In the study, we focused on the analysis of optical coherence tomography (OCT) images of the cornea and retina including corneal thickness, central retinal thickness, retinal nerve fiber layer (RNFL), and optic disc parameters. The measurements were acquired using Spectral-domain OCT REVO FC 130. **Results:** The analysis did not show significant changes between the examined ophthalmological parameters before and after therapy. Furthermore, there were no detected significant differences between the tested parameters of the retina and cornea in COVID-19-positive patients compared to the control group. **Conclusions:** No ophthalmological manifestations of COVID-19 disease were observed during the study. Taking into account the results of other publications, the lack of an unambiguous position on this topic requires further research.

## 1. Introduction

According to the latest WHO updates, COVID-19, an infectious respiratory disease triggered by the SARS-CoV-2 virus, has caused over 700 million infections and over 7 million deaths worldwide since the first cases appeared at the end of 2019 [1]. Due to its global impact and rapid pandemic expansion, this disease continues to pose challenges and drive further research. The range of possible symptoms of SARS-CoV-2 virus infection is wide, ranging from minimal parainfluenza symptoms to acute respiratory distress syndrome. Additionally, there is a risk of multi-organ complications, including cardiovascular, neurological, gastrointestinal, and hematological issues [2,3]. Since the onset of the pandemic, there has been an increasing number of publications indicating ocular manifestations of the disease. The most frequently described ophthalmological symptoms include ailments related to the anterior segment of the eye, such as conjunctivitis, eye pain, dry eye, foreign body sensation, tearing, and redness [4,5,6]. Furthermore, studies suggest the possibility of retinal pathology occurring during COVID-19. One of the described retinal changes is microangiopathy with cotton wool spots [7]. Some publications based on OCT results suggest changes in the optic nerve fiber layer and ganglion cell layer [8]. Most studies involve patients who have recovered from COVID-19 rather than those with active infection. This study aims to assess changes in the morphology of the retina and cornea in patients treated and hospitalized during the acute active phase of SARS-CoV-2 infection. In this research we relied on OCT, which is an excellent device for monitoring changes in both the retina and the cornea. OCT is a powerful non-invasive optical imaging modality that allows for in vivo structural and functional imaging of the retina and cornea of humans and experimental animals with cellular-level resolution [9,10].

## 2. Materials and Methodology

The study was carried out at the International Congress Center in Katowice, which was temporarily transformed into a field hospital for COVID-19 patients, spanning from December 2020 to May 2021. Approval was obtained from the Bioethics Committee of the University of Technology in Katowice 01/KEBN/2021on 16 July 2021. The study included 24 participants (14 men and 10 women) aged between 30 and 78 years, with a mean age of 56.125 ± 12.895. All participants had symptomatic SARS-CoV-2 infection confirmed by a positive PCR test of the nasopharyngeal swab. The study was conducted during the hospitalization of patients in the acute phase of severe COVID-19 requiring oxygen therapy. The general inclusion criteria encompassed stable hemodynamic status, ability to provide informed consent, hospitalization during the acute active phase, and a confirmed diagnosis of SARS-CoV-2 infection, while ophthalmic exclusion criteria comprised media opacities, fixation loss, presence of glaucoma, intraocular pressure (IOP) exceeding 21 mmHg, corneal epithelium abnormalities such as scars or dystrophies, as well as age-related macular degeneration (AMD) and retinal dystrophies. General medical exclusion conditions included diabetes of any type, uncontrolled hypertension, coagulations and blood disorders, and mechanical ventilation. 

**Figure 1:**
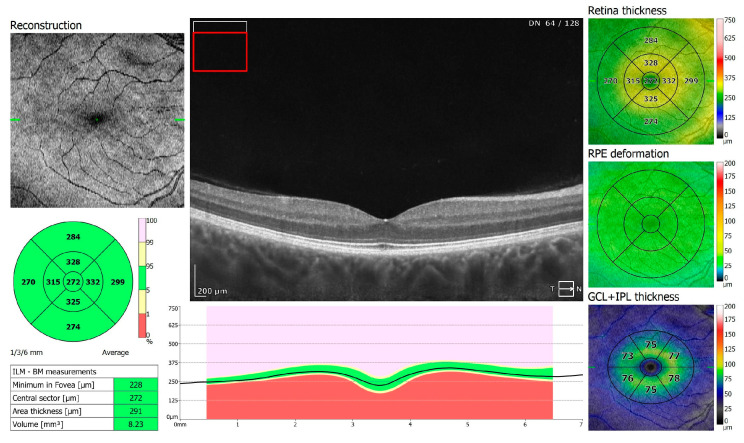
OCT 3D macula scan 7 × 7 mm of COVID-19-positive patient. RPE, retinal pigment epithelium; GCL + IPL, ganglion cells layer + inner plexiform layer; ILM–BM, inner limiting membrane–Bruch’s membrane.

**Figure 2:**
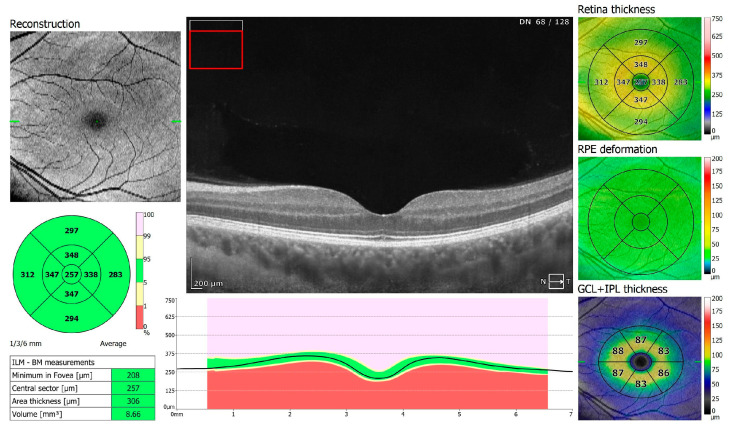
OCT 3D macula scan 7 × 7 mm of COVID-19-negative patient. RPE, retinal pigment epithelium; GCL + IPL, ganglion cells layer + inner plexiform layer; ILM–BM, inner limiting membrane–Bruch’s membrane.

**Figure 3:**
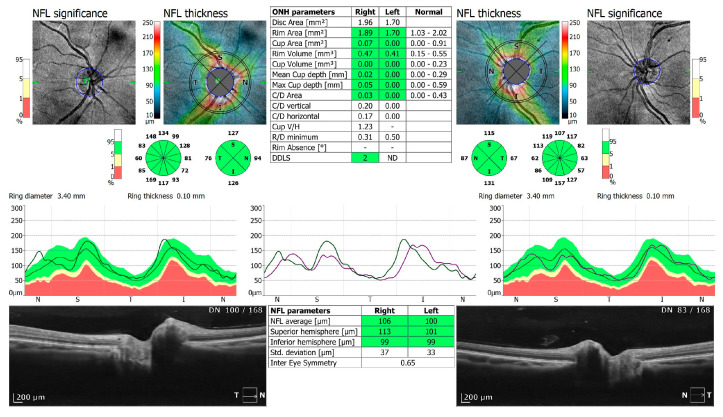
OCT 3D disc scan 6 × 6 mm of COVID-19-positive patient. NFL, nerve fiber layer; ONH, optic nerve head.

**Figure 4:**
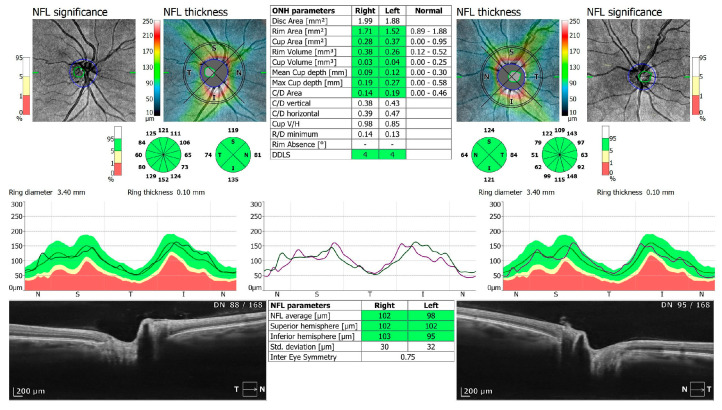
OCT 3D disc scan 6 × 6 mm of COVID-19 negative patient. NFL, nerve fiber layer; ONH, optic nerve head.

**Figure 5:**
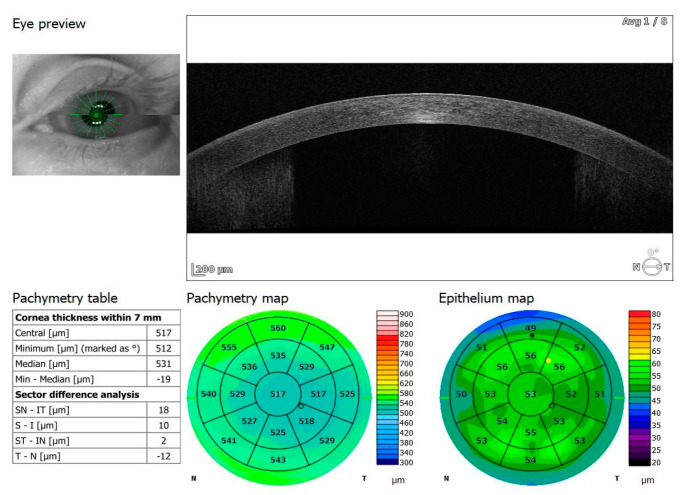
OCT anterior radial scan 8 × 8 mm of COVID-19 positive patient.

**Figure 6:**
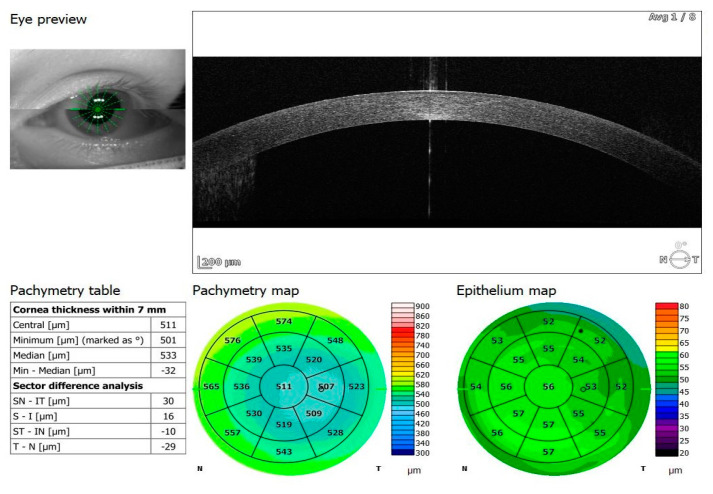
OCT anterior radial scan 8 × 8 mm of COVID-19-negative patient.

Of the participants, 20 received oxygen therapy at flow rates ranging from 1–10 L, while 4 received high-flow intranasal oxygen therapy (HFNOT). Additionally, 5 patients were treated with Remdesivir therapy, 5 received COVID-19 convalescent plasma therapy, and 11 were treated with Tocilizumab. The demographics and clinical characteristics of the study participants are presented in Table 1. The mean time from a positive PCR test to study enrollment was 13.23 days, with a standard deviation of 8.18 days. The control group comprised 38 healthy individuals: 28 men and 10 women (control versus research group: *p* = 0.21), with an average age of 58 years, ranging from 33 to 69 years. All participants underwent two examinations, one upon admission and another upon cessation of therapy (6.70 ± 6.46 days). Spectral-domain optical coherence tomography (SD-OCT) was conducted using REVO FC 130 (Optopol Technology, Zawiercie, Poland). This device allows for high-resolution imaging and quantitative analysis of both the retina and cornea. The study specifically evaluated central retinal thickness, retinal nerve fiber layer (RNFL) thickness and optic disc parameters, alongside central corneal thickness and corneal epithelium thickness. The measurements were performed using built-in retinal and corneal layer segmentation tools provided by the OCT system, which automatically segmented the different layers of the retina and cornea to ensure precise and repeatable thickness measurements. OCT protocols included a 3D macula 7 × 7 mm scan, 3D disc 6 × 6 mm scan, and anterior radial 8 × 8 mm scan (Figure 1, Figure 2, Figure 3, Figure 4, Figure 5 and Figure 6). All patients had also undergone slit lamp examination, including indirect ophthalmoscopy.

### Statistical Analysis

Statistical calculations were made using the Statistica v. 13.3 software (Tibco, Palo Alto, CA, USA). The normality of the data was checked using the Shapiro–Wilk test, and non-parametric data were presented in the form of medians, with the minimum and maximum values presented. To avoid bias, only one randomly selected eye was used for analysis. Repeated ANOVA was used to examine changes between groups. Oxygen therapy was divided into two groups, one with 1–10 L flow and the HFNOT. Correlation was used to evaluate between the measured ocular parameters, and a *p*-value of less than 0.05 was considered statistically significant.

## 3. Results

Repeated analysis of variance ANOVA did not show significant changes between the examined ophthalmological parameters before and after therapy (Table 2). The study also did not detect significant differences between the tested parameters of the retina and cornea in COVID-19-positive patients compared to the control group (Table 2). 

In the study, we measured the correlation between the individual parameters (Table 3). The analysis showed no statistically significant correlations between all examined parameters except age and RNFL thickness (r = −0.76, *p* < 0.05) and age and corneal epithelium thickness (r = −0.90, *p* < 0.05). The study did not detect a statistically significant correlation between pachymetry results and plasma treatment (*p* = 0.65), oxygen therapy with different settings (*p* = 0.48), remdesivir (*p* = 0.85), or tocilizumab (*p* = 0.21). 

Similarly, there was no statistically significant correlation between RNFL thickness and treatment with plasma (*p* = 0.49), oxygen therapy with different settings (*p* = 0.39), remdesivir (*p* = 0.49), or tocilizumab (*p* = 0.56).

Moreover, the study did not show a statistically significant correlation between the cup-to-disc ratio and treatment with plasma (*p* = 0.40), oxygen therapy with different settings (*p* = 0.10), remdesivir (*p* = 0.98), or tocilizumab (*p* = 0.99).

The analysis also did not detect a statistically significant correlation between central macular thickness and treatment with plasma (*p* = 0.38), HFNOT (*p* = 0.21), remdesivir (*p* = 0.23), and tocilizumab (*p* = 0.15). Slit lamp examination including indirect ophthalmoscopy did not reveal any signs of new pathology during infection. 

## 4. Discussion

Our study, conducted amidst the COVID-19 pandemic, found no significant changes in ocular parameters between healthy individuals and those infected. Additionally, we observed no significant alterations in the impact of therapy on ocular parameters.

Since the emergence of the first identified case of SARS-CoV-2 infection in December 2019 in Wuhan, China, there has been extensive research on the virus’s potential impact on ocular health. However, despite this observation, a clear consensus on the potential eye complications of COVID-19 remains elusive. Conjunctivitis and its associated symptoms have emerged as among the most frequently documented manifestations involving the anterior segment of the eye [5]. In the course of COVID-19, patients may also develop other ocular surface diseases, including keratitis, pseudomembranous keratoconjunctivitis, or episcleritis. However, so far, these cases have been presented mainly as clinical case reports [11,12,13,14]. The range of published possible ophthalmological complications in the course of SARS-CoV-2 infection is wide, but it has not yet been determined whether they are a result of the disease or a complication of the therapy [15].

Corneal pachymetry is influenced by various factors, with the condition of endothelial cells playing a crucial role in maintaining proper hydration of the corneal parenchyma through the sodium-potassium pump [16]. Erdem et al. and Oren et al. have reported a decrease in endothelial cell count and an increase in CCT shortly after COVID-19 infection compared to control groups. Oren emphasized that while CCT changes were not statistically significant, the reduced endothelial cell density remained within the normal range. They suggested a potential link between increased corneal thickness and endothelial dysfunction due to immune dysregulation and pro-inflammatory activity during infection [15,17]. Similarly, Ismail’s analysis also noted a reduction in endothelial cell count without significant changes in CCT or corneal epithelial thickness [18].

In our previous study evaluating various eye surface parameters, we found no statistically significant differences between post-COVID-19 patients and controls [19]. Specifically, our analysis using a different machine, focused on CCT and corneal epithelial thickness, revealed no significant correlations with other examined parameters except for age and corneal epithelial thickness. This relationship is consistent with the opinion that the thickness of the corneal epithelium decreases with age, which is also confirmed by another study [20]. We also observed no significant differences in corneal parameters between patients before and after therapy, or between patients and controls, which aligns with findings by Ornek et al., who found no significant changes in pachymetry results between hospitalized COVID-19 patients and controls [21].

It is notable that unlike most studies, Ornek et al.’s research and ours involved patients in the active phase of COVID-19 rather than during convalescence or post-COVID-19. This temporal difference could account for variations in results, as the short duration of exposure to the infectious agent might limit the observation of corneal changes.

As the pandemic progressed, an increasing number of studies investigating the impact of the SARS-CoV-2 virus on the posterior segment of the eye emerged. Indeed, retinal involvement in COVID-19 appears to be rare compared to ocular surface diseases. Typically, such changes are characterized as subtle retinopathy, featuring cotton wool spots, microhemorrhages, or flame-shaped hemorrhages, along with vessel dilatation and tortuosity [22,23,24,25]. However, it is important to note that studies reporting these cases often did not exclude patients with concomitant diseases such as diabetes or hypertension. Consequently, it is challenging to discern to what extent observed fundus changes were directly caused by the virus or were manifestations of retinopathy associated with concurrent conditions. This concern was raised by Vavvas et al., citing the work of Marinho et al. [22], and questioning the validity of this analysis, particularly due to the lack of exclusion of comorbidities [26]. Among the literature, reports indicate no fundus changes or no impact on certain OCT parameters in patients following COVID-19 [27,28]. 

Reports on retinopathy in the context of COVID-19 remain inconclusive, with various potential mechanisms proposed for the virus’s impact on optic disc morphology. One theory suggests direct nervous system involvement due to the neurotropic nature of the SARS-CoV-2 virus [29], often manifesting as neurological symptoms such as anosmia and ageusia. Burgos-Blasco et al. found a statistically significant association between increased peripapillary retinal nerve fiber layer (pRNFL) thickness in recovered patients and symptoms of smell and taste disorders [30]. Another potential mechanism involves the influence of various therapies, particularly anti-inflammatory treatments. Additionally, the virus may induce inflammation and transient ischemia in the peripapillary vessel endothelium, leading to edema, ischemia, and subsequent optic nerve atrophy. Savastano et al. described microcirculation damage by evaluating angio-OCT parameters and RNFL in patients post-COVID-19 [31]. Coagulation disorders, commonly observed in SARS-CoV-2 infection, especially in acute cases, may render patients more susceptible to thromboembolic events affecting both veins and arteries, potentially resulting in central retinal vein occlusion or central retinal artery occlusion [32,33].

As mentioned earlier, publications on changes in OCT parameters of the retina and optic disc during COVID-19 present ambiguous results due to differences in study methodology, selection of research and control groups, study duration, and time from diagnosis to analysis. Inclusion and exclusion criteria are also significant, including the presence of comorbidities and the therapy used. In our study, we assessed OCT changes in patients during the acute phase of COVID-19 hospitalization for a relatively short period. However, we lacked comparisons with results during convalescence or before disease onset. Bayram et al. studied patients hospitalized with SARS-CoV-2 pneumonia in the active acute phase, observing significantly greater peripapillary retinal nerve fiber thickness compared to other groups. Conversely, Abrishami et al. demonstrated a lack of statistical significance in increased RNFL thickness in all sectors of the optic disc, possibly influenced by steroid therapy [34,35]. Subsequent work by Abrishami et al. revealed significant local pRNFL loss in the inferonasal sector three months post-disease, while optic nerve disc morphology parameters remained unchanged [36]. Similarly, in our study, we found no statistically significant correlation between examined parameters of the retina and optic disc, except for age and RNFL thickness, as well as the number of days since COVID-19 diagnosis and RNFL thickness. The former correlation can be explained by the natural decrease in pRNFL thickness with age [37]. Comparing the results of sick patients with the results of the control group, we showed no significant differences in the examined parameters of the retina and optic disc. Similarly, we did not demonstrate significant differences in these parameters when comparing sick people before and after COVID-19 therapy. In contrast to our analysis, Javnikar et al. demonstrated an increase in RNFL thickness in the upper and lower quadrants in patients hospitalized due to the acute phase of COVID-19 [38]. Kal et al. conducted a study two months after discharge of patients hospitalized due to COVID-19 and found no optic disc edema, as well as no statistically significant changes in cup-to-disc parameters and peripheral area in the research and control groups [28]. However, this contradicts the results of the same authors’ earlier study, which described the thickening of the RNFL in such patients [39]. Seker et al. showed statistically significant thinning of the pRNFL in patients examined during convalescence after COVID-19 without prior hospitalization [40]. Conversely, our study found no changes in central macular thickness and RNFL parameters in patients during the early post-COVID period compared to the control group [27].

The purpose of our analysis was to assess the impact of COVID-19 therapy on OCT parameters of the anterior and posterior segments of the eye. The study participants underwent various types of therapy, including oxygen therapy, primarily recommended for severe COVID-19 cases. The WHO recommends different kinds of oxygen therapy, from low to high flow rates. Additionally, oxygen can be delivered in higher concentrations using high-flow nasal oxygen therapy (HFNOT). Moreover, a very acute course of the disease may require invasive ventilation devices [41]. In our study, most patients received oxygen therapy with a flow of 1–10 L, while others were treated with HFNOT. We did not find publications about ocular complications caused by oxygen therapy, except for ocular surface manifestations, such as dry eye or conjunctivitis. Ceylan et al. indicated an increased risk of ocular surface complications in patients treated with HFNOT; most often, the symptoms of ocular surface damage were related to mechanical ventilation and the need for sedation [42]. However, in our study, where most patients received conventional oxygen therapy and were not mechanically ventilated, we did not observe serious ocular surface complications influencing pachymetry parameters.

Another therapy used was remdesivir, an adenosine triphosphate (ATP) analog metabolized to remdesivir triphosphate (RDV-TP) intracellularly, which inhibits SARS-CoV-2 RNA-dependent RNA polymerase. Timothy P.H. Lin et al. found no ocular side effects of remdesivir [43]. In our study, we did not find a correlation between remdesivir use and OCT parameters, nor did we find studies assessing its effect on OCT findings.

Tocilizumab, an IL-6 receptor antagonist, showed promise in reducing mortality and the need for mechanical ventilation among COVID-19 patients [44,45,46]. In our study, we found no correlation between tocilizumab therapy and OCT parameters, nor did we find studies suggesting an effect of this therapy on OCT findings.

Plasma therapy from COVID-19 convalescents, containing neutralizing antibodies against SARS-CoV-2 spike protein, did not show significant improvement in clinical outcomes [47]. We found no correlation between plasma therapy and changes in OCT, nor did we find reports on its impact on OCT findings.

There are publications on the impact of preventive behaviors on pachymetry parameters. Zein et al. found no statistically significant changes in corneal topography and tomography parameters, including CCT, in patients wearing protective masks compared to those who did not [48].

Among the limitations of our study, we can include a lack of data on the patients before the infections, a relatively small sample size, and a lack of mechanically ventilated patients. 

Our ability to compare results with other studies was hindered by a lack of research on the effects of different COVID-19 therapies on OCT changes. Further investigations are essential, particularly with larger sample sizes, pre-disease OCT data for comparison, extended observation periods, and exclusion of comorbidities. Such studies can provide valuable insights into conducting eye examinations for COVID-19 patients and determining optimal therapeutic approaches. Given the ongoing impact of the COVID-19 pandemic, advancing our understanding of its effects on ocular health is paramount.

## 5. Conclusions

In conclusion, our study did not identify ophthalmological manifestations of COVID-19 disease. Given the diversity of research on OCT changes in this infection, it remains challenging to determine whether SARS-CoV-2 is the primary cause of these alterations. We found no correlation between various COVID-19 therapies and changes in OCT parameters of the retina and cornea in affected patients. Antiviral therapy did not change the parameters in the studied group, but it should be noted that a low number of patients may have biased the findings. Furthermore, we observed no clinically significant differences in tested parameters between the research and control groups, nor did we detect changes before and after COVID-19 therapy. It is worth considering whether the absence of ocular manifestations in OCT among patients undergoing therapy may be attributed to its therapeutic efficacy.

## Figures and Tables

**Table 1:** Demographics and clinical characteristics of participants.

	Number of Participants	
	COVID-19 +	COVID-19 −
**Male**	14	28
**Female**	10	10
**Oxygen therapy 1–10 L**	20	
**HFNOT**	4	
**Remdesivir:**		
Yes	5	
No	19	
**Tocilizumab:**		
Yes	11	
No	13	
**Convalescent plasma:**		
Yes	5	
No	19	
**Average duration of the therapy**	6.70 (range: 2–35 days)	

**Table 2:** Repeated ANOVA results.

Variable	COVID-19 before Treatment				
	Median	Minimum	Maximum	Standard	*p* Value vs. COVID-19 after Treatment
Macular Volume	7.74	7.13	8.79	0.06	0.63
Central Macular Thickness	232.00	195.00	303.00	3.47	0.81
Central Corneal Thickness	541.00	471.00	704.00	6.30	0.88
Mean RNFL thickness	104.00	85.00	124.00	1.31	0.48
Corneal Epithelium	57.00	48.00	72.00	0.96	0.45
Age	58.00	33.00	69.00	3.30	NA
Average Macular Thickness	274.00	252.00	311.00	1.99	0.84
Variable	COVID-19 after treatment				
	Median	Minimum	Maximum	Standard	NA
Macular Volume	7.70	7.24	8.82	0.06	NA
Central Macular Thickness	233.00	212.00	293.00	3.13	NA
Central Corneal Thickness	534.00	495.00	586.00	4.10	NA
Mean RNFL thickness	104.00	85.00	122.00	1.53	NA
Corneal Epithelium	57.00	51.00	70.00	1.12	NA
Age	61.00	30.00	78.00	4.44	NA
Average Macular Thickness	272.00	256.00	312.00	2.04	NA
Variable	group = Control Descriptive Statistics (Spreadsheet z control)				
	Median	Minimum	Maximum	Standard	*p* value (vs. the COVID-19 group)
Macular Volume	7.74	7.24	8.76	0.05	0.93
Central Macular Thickness	229.00	203.00	266.00	2.36	0.95
Central Corneal Thickness	537.00	480.00	648.00	5.76	0.16
Mean RNFL thickness	104.00	76.00	130.00	1.61	0.90
Corneal Epithelium	57.00	52.00	67.00	0.59	0.56
Age	61.00	40.00	79.00	1.89	0.43
Average Macular Thickness	275.00	232.00	316.00	2.85	0.83

**Table 3:** Correlation matrix for tested parameters. Significant correlations (*p* < 0.05) are marked in red.

	Oxygen Therapy (L/min)	Oxygen Therapy Settings	Length of COVID-19	Remdesivir	Plasma	Tocilizumab
Gender	−0.24	−0.25	−0.30	0.05	−0.05	−0.22
Age	0.33	0.28	0.30	0.06	−0.07	−0.24
Central Macular Thickness	0.30	0.16	0.07	−0.12	−0.25	0.45
Average retinal thickness	0.16	0.05	0.10	−0.37	−0.31	0.37
Macular Volume	0.16	0.05	0.10	−0.37	−0.30	0.38
Disc size	0.39	0.28	−0.01	−0.30	−0.19	−0.06
Rim	0.18	0.21	0.00	−0.14	−0.12	0.53
Cup	0.10	−0.00	−0.00	−0.07	−0.02	−0.52
Mean cup	−0.09	−0.19	0.03	−0.03	0.07	−0.37
c/d ratio	−0.00	−0.09	−0.02	−0.03	0.00	−0.56
Mean RNFL thickness	0.37	0.38	0.26	−0.34	−0.23	0.51
Central corneal thickness	−0.16	−0.10	−0.02	−0.40	−0.38	0.11
Corneal epithelium center	−0.42	−0.47	−0.06	0.38	0.28	0.01

See Supplementary Table S1 for list of all correlations.

## Data Availability

The dataset is available on request from the authors.

## Supplementary Materials

The following supporting information can be downloaded at: https://www.mdpi.com/article/10.3390/jcm13185564/s1, Table S1: Correlation matrix for tested parameters.
