# Supplementary material for: Retinal and Corneal OCT Results of Patients Hospitalized and Treated in the Acute Phase of COVID-19
Source: J Clin Med. 2024 Sep 19;13(18):5564. doi: 10.3390/jcm13185564 (PMC11432357; doi:10.3390/jcm13185564)
Supplement: Supplementary file 1 [file jcm-13-05564-s001.zip › jcm-3094231-supplementary.pdf]

Table S1: Correlation matrix for tested parameters.

	Oxygen therapy (l/min)	Oxygen therapy settings	Length of COVID-19	Remdesivir	Plasma	Tocilizumab
Gender	-0.24	-0.25	-0.30	0.05	-0.05	-0.22
Age	0.33	0.28	0.30	0.06	-0.07	-0.24
Central Macular Thickness	0.30	0.16	0.07	-0.12	-0.25	0.45
Retinal thickens 2	0.11	0.05	-0.05	-0.47	-0.32	0.26
Retinal thickens 3	0.20	0.14	-0.01	-0.42	-0.28	0.32
Retinal thickens 4	0.17	0.11	-0.09	-0.47	-0.34	0.24
Retinal thickens 5	0.10	0.03	-0.14	-0.45	-0.36	0.27
Retinal thickens 6	-0.00	-0.09	0.13	-0.06	-0.05	0.13
Retinal thickens 7	0.25	0.19	0.12	-0.43	-0.28	0.33
Retinal thickens 8	0.19	0.04	0.13	-0.33	-0.30	0.42
Retinal thickens 9	0.11	0.00	0.15	-0.42	-0.41	0.51
Minimal	0.22	0.12	0.00	-0.13	-0.24	0.35
centrum	0.30	0.16	0.07	-0.12	-0.25	0.45
Average retinal thickness	0.16	0.05	0.10	-0.37	-0.31	0.37
Volume	0.16	0.05	0.10	-0.37	-0.30	0.38
RNFL S	-0.02	-0.05	0.01	-0.13	-0.12	0.31
RNFL N	0.26	0.30	0.18	-0.23	-0.04	0.37
RNFL I	0.59	0.61	0.40	-0.46	-0.32	0.51
RNFL T	0.03	0.01	0.03	0.25	0.02	-0.03
RNFL 1	-0.10	-0.14	-0.01	-0.14	-0.09	0.20
RNFL 2	0.19	0.14	-0.00	-0.21	-0.09	-0.00
RNFL 3	0.10	0.12	-0.14	-0.26	0.02	0.26
RNFL 4	0.40	0.44	0.43	-0.16	-0.09	0.42
RNFL 5	0.61	0.58	0.32	-0.49	-0.32	0.43
RNFL 6	0.35	0.43	0.28	-0.50	-0.27	0.38

RNFL 7	-0.10	-0.10	0.05	0.35	0.10	-0.01
RNFL 8	0.02	0.02	0.08	0.31	0.13	-0.11
RNFL 9	0.04	0.01	-0.03	0.17	-0.03	0.01
RNFL 10	-0.01	-0.00	0.05	0.05	-0.05	0.27
Disc size	0.39	0.28	-0.01	-0.30	-0.19	-0.06
Rim	0.18	0.21	0.00	-0.14	-0.12	0.53
Cup	0.10	-0.00	-0.00	-0.07	-0.02	-0.52
mean cup	-0.09	-0.19	0.03	-0.03	0.07	-0.37
c/d ratio	-0.00	-0.09	-0.02	-0.03	0.00	-0.56
mean RNFL thickness	0.37	0.38	0.26	-0.34	-0.23	0.51
Central corneal thickness	-0.16	-0.10	-0.02	-0.40	-0.38	0.11
corneal thickness 2	-0.20	-0.18	-0.26	-0.28	-0.25	-0.14
corneal thickness 3	-0.13	-0.10	0.02	-0.44	-0.37	0.00
corneal thickness 4	-0.10	-0.06	0.11	-0.46	-0.40	0.12
corneal thickness 5	-0.12	-0.06	0.03	-0.42	-0.37	0.14
corneal thickness 6	-0.26	-0.15	-0.26	-0.08	-0.10	0.03
corneal thickness 7	-0.29	-0.19	-0.33	-0.04	-0.07	-0.00
corneal thickness 8	-0.27	-0.20	-0.26	-0.23	-0.23	0.03
corneal thickness 9	-0.26	-0.21	-0.25	-0.31	-0.27	-0.06
Corneal epithelium center	-0.42	-0.47	-0.06	0.38	0.28	0.01
Corneal epithelium 2	-0.05	0.00	0.24	-0.37	-0.19	0.17
Corneal epithelium 3	-0.31	-0.25	-0.16	0.25	0.28	-0.02
Corneal epithelium 4	0.16	0.18	0.32	0.01	-0.20	0.03
Corneal epithelium 5	0.35	0.43	0.174	-0.46	-0.40	0.32
Corneal epithelium 6	-0.03	-0.02	-0.29	-0.20	-0.10	-0.20
Corneal epithelium 7	-0.08	-0.21	-0.50	-0.21	-0.22	-0.30
Corneal epithelium 8	-0.24	-0.28	-0.09	-0.21	0.06	-0.13
Corneal epithelium 9	-0.25	-0.30	-0.17	0.41	0.55	-0.06
